# Normal Tail Suppression for Low-False-Positive RGB–3D Industrial Anomaly Localization

**DOI:** 10.3390/jimaging12100466

**Published:** 2026-09-24

**Authors:** Guiping Zhu, Zhenyan Ji, Zhentao Wu, Jiuqian Dai, Wenhui Chen, Hui Liu

**Affiliations:** 1 School of Software Engineering, Beijing Jiaotong University, Beijing 100044, China; 22110144@bjtu.edu.cn (G.Z.); 23111495@bjtu.edu.cn (J.D.); wenhuichen@bjtu.edu.cn (W.C.); 24110480@bjtu.edu.cn (H.L.); 2 School of Cyberspace Science and Technology, Beijing Jiaotong University, Beijing 100044, China; 3 National College for Excellent Engineers, Beijing Jiaotong University, Beijing 100044, China; wu.zhentao@bjtu.edu.cn

**Keywords:** industrial anomaly detection, multimodal anomaly detection, false-alarm-aware localization, normal tail suppression

## Abstract

RGB images and 3D point clouds provide complementary appearance and geometric cues for industrial anomaly localization, yet a small number of high-scoring normal regions can still produce spurious defect candidates under stringent false-positive constraints. We propose Normal Tail Suppression (NTS), a training-stage regularizer that selectively penalizes the upper tail of normal fused scores while leaving the deployed inference architecture unchanged. By concentrating optimization on the normal responses most relevant to low-false-positive-rate (FPR) operation, NTS avoids uniformly shrinking the normal-score distribution. On MVTec 3D Anomaly Detection (MVTec 3D-AD), NTS improves mean AUPRO@1% by +0.352 percentage points over the matched control and maintains a positive mean trend across five paired seeds. Sensitivity, control-ablation, threshold-resolution, and score-distribution analyses support the intended tail-focused behavior, while a protocol-frozen second fusion configuration provides further positive evidence. These results establish NTS as a simple, deployment-compatible mechanism for improving low-FPR RGB–3D anomaly localization within the evaluated configurations.

## 1. Introduction

Multimodal industrial anomaly detection combines complementary sensing channels for visual inspection [1,2,3,4,5]. RGB images capture appearance, texture, and color irregularities, while 3D point clouds describe geometry, depth, and structural deformation that may be difficult to identify from RGB images alone. Since the release of the MVTec 3D Anomaly Detection (MVTec 3D-AD) benchmark [1], representative methods have exploited RGB–3D complementarity through different mechanisms: Asymmetric Student–Teacher (AST) [6] combines a normalizing-flow teacher with an asymmetric student, Multi-3D-Memory (M3DM) [2] employs hybrid fusion with multiple memory banks, Crossmodal Feature Mapping (CFM) [4] learns mappings between RGB and 3D representations, and Geometry-Guided Score Fusion ($G^{2}$SF) [5] learns geometry-aware metrics for multimodal score fusion. Despite these advances, practical inspection systems may also require reliable anomaly localization under stringent false-positive constraints.

In practical industrial inspection, anomaly maps are often used to direct attention toward candidate defect regions. Ideally, genuine defects should produce salient responses, while normal surface variations remain at low scores. In practice, however, a small number of normal regions may receive high anomaly scores and appear as spurious defect candidates [5,7]. Reliable localization under stringent false-positive constraints is therefore important for maintaining the usability of automated inspection systems. We evaluate this operating regime using the area under the per-region-overlap curve (AUPRO) up to a 1% false-positive rate (FPR), denoted AUPRO@1% [5,8].

Such spurious responses are particularly relevant in RGB–3D inspection because the two modalities are affected by different forms of normal variation [2,3,5]. Surface texture, reflectance, and illumination changes may produce unusual RGB features, while sparse or missing point-cloud observations can create unstable geometric responses. Object boundaries and modality-specific uncertainty may also be amplified during multimodal score fusion, causing some normal regions to receive disproportionately high fused anomaly scores. Existing RGB–3D methods address this problem only indirectly: AST adopts asymmetric student–teacher learning [6], M3DM constructs multimodal feature memories [2], CFM learns cross-modal feature mappings [4], and $G^{2}$SF combines geometry-guided metric learning with score fusion [5]. Beyond RGB–3D detection, Hard-normal Example-aware Template Mutual Matching (HETMM) models hard-normal samples that may be confused with anomalies [7], Online Hard Example Mining (OHEM) emphasizes difficult labeled examples during supervised detector training [9], and conditional value-at-risk (CVaR) optimization focuses on the upper tail of a loss distribution [10]. In the setting considered here, however, these approaches do not directly isolate and regularize the upper tail of normal fused scores during RGB–3D fusion training. An objective that explicitly suppresses these high-response normal entries without adding inference-time parameters, modules, or state therefore remains worth investigating.

We therefore study Normal Tail Suppression (NTS), where the primary implementation adopts a batchwise tail estimator, as a training-stage intervention that selects and penalizes high-response trainer-labeled normal fused-score training entries. Rather than introducing an additional inference module, the NTS regularizer modifies fusion-stage optimization by selecting the upper tail of normal scores within each mini-batch. NTS does not assume that the normal upper tail is the sole limitation of anomaly localization, but examines whether adding this loss term is associated with improved low-FPR localization under a matched RGB–3D continuation and fixed-final checkpoint protocol.

The main contributions are summarized as follows:

1. We formulate high-scoring normal regions as a practical source of spurious defect candidates in RGB–3D industrial inspection and use the upper tail of normal fused training scores as an actionable proxy for localization under stringent false-positive constraints.
2. We propose Normal Tail Suppression (NTS), a training-stage regularizer that selects and penalizes the top-*q* trainer-labeled normal fused-score entries within each mini-batch. It introduces no additional inference-time parameters, modules, or state and leaves the deployed anomaly-localization pipeline unchanged.
3. On MVTec 3D-AD, under a matched fixed-final continuation protocol, NTS obtains a pipeline-reported ten-category mean AUPRO@1% of 47.494%, a paired difference of +0.352 percentage points over the matched control without NTS (47.142%), with a positive mean trend across five paired seeds. This evidence is bounded to the tested continuation protocol, and the published $G^{2}$SF value of 46.8% is cited only as non-matched context.

## 2. Related Work

### 2.1. Multimodal Industrial Anomaly Detection

Unsupervised industrial detection generally models the distribution of defect-free samples because anomalous data are rare, diverse, and expensive to collect [8,11]. Representative RGB-based methods include distribution-modeling approaches such as Patch Distribution Modeling (PaDiM) [12] and memory-bank retrieval approaches such as PatchCore [13], illustrating the effectiveness of pretrained visual features and patch-level normal representations for anomaly detection and localization [14]. EfficientAD further shows that efficient student–teacher designs can also achieve strong visual anomaly detection performance with low latency [15]. Building on this foundation, the release of the RGB–3D MVTec 3D-AD benchmark [1] extended industrial detection to complementary appearance and geometric information: RGB images characterize texture and color variations, whereas 3D point clouds capture depth and structural deformation [2,3]. Recent multimodal methods therefore extract modality-specific representations from pretrained image and point-cloud backbones and combine their anomaly evidence for more comprehensive defect detection [2,3,4,5].

Representative RGB–3D methods exploit cross-modal complementarity through different learning and fusion strategies [2,3]. Related 3D anomaly detection studies also explore alternative matching-based designs [16]. AST introduces an asymmetric student–teacher framework for industrial anomaly detection [6], while M3DM combines point-feature alignment, multiple memory banks, and decision-level fusion [2]. Shape-guided dual-memory learning employs specialized color and shape experts to integrate appearance and geometric information [17], whereas CFM learns cross-modal mappings from nominal samples and identifies anomalies through the difference between predicted and mapped features [4]. CPMF constructs pseudo-multimodal representations from point-cloud-derived handcrafted 3D descriptors and projected pseudo-2D views [18]; it is therefore distinct from methods that fuse independently acquired RGB and 3D observations. More recent studies extend these efforts through memoryless multimodal detection [19], multimodal reverse distillation [3], and geometry-guided score fusion [5] to improve efficiency and modality interaction. Among them, $G^{2}$SF is most closely related to the present work: it preserves modality-specific anomaly evidence and combines RGB and 3D scores through geometry-guided fusion, thereby addressing overestimated responses on normal regions indirectly [5]. Building on this pipeline, NTS targets these responses explicitly by using a top-*q* rule to select and penalize high-response trainer-labeled normal fused-score entries during training.

### 2.2. Low-FPR Anomaly Localization and Hard-Normal Responses

Most industrial anomaly detection studies report broad-range metrics such as image-level area under the receiver operating characteristic curve (I-AUROC), pixel-level area under the receiver operating characteristic curve (P-AUROC), and AUPRO@30% [1,5,8,15]. Partial AUC characterizes performance within a specified FPR interval [20], while restricted-FPR per-region overlap (PRO)-based summaries provide a focused characterization of low-FPR anomaly localization [5,17]. However, evaluation alone cannot reduce the high-scoring normal responses that produce spurious defect candidates, making training-stage modeling necessary. HETMM learns from hard-normal examples that may be confused with anomalies [7], whereas $G^{2}$SF indirectly reduces overestimated normal responses through geometry-guided metric learning and score fusion [5]. Neither method explicitly isolates and regularizes the upper tail of normal RGB–3D fused training scores. NTS addresses this limitation by applying a top-*q* rule to select and penalize current-batch normal fused-score training entries as training proxies for possible spurious defect responses during fusion training.

### 2.3. Tail-Focused and Risk-Aware Optimization

Hard-example mining and tail-focused optimization provide relevant foundations for emphasizing difficult training responses. OHEM selects difficult labeled examples during supervised detector training [9], average top-*k* objectives optimize the largest sample losses [21], and CVaR risk minimization emphasizes the upper tail of a loss distribution [10]. However, these formulations operate on labeled hard examples or generic loss distributions and do not directly specify how to handle unusually high normal fused scores in normal-only RGB–3D anomaly training, where labeled real defects and conventional hard negatives are unavailable. NTS adapts the tail-focused principle to this setting by applying a top-*q* rule to select high-response normal fused-score entries from the current mini-batch and penalizing their mean. Because the primary NTS implementation uses only current-batch scores, it requires no cross-batch estimator state and adds no parameters, modules, or computation to the deployed inference path. This design directly targets problematic normal responses while preserving the original RGB–3D localization pipeline, although it does not introduce new top-*k* or CVaR theory. NTS applies an average-top-*k*-type penalty to high-response trainer-labeled normal fused scores in normal-only RGB–3D anomaly training. It uses no labeled hard negatives, requires no cross-batch state in its primary form, and does not introduce new CVaR theory.

## 3. Methodology

### 3.1. Method Overview

The proposed framework retains the main stages of the $G^{2}$SF RGB–3D anomaly localization pipeline [5]: frozen RGB and 3D backbones extract appearance and geometric representations, modality-specific coreset memories and nearest-neighbor retrieval produce anomaly evidence, and a fusion scorer combines the two modalities into fused patch scores. The patch scores are subsequently upsampled and smoothed to generate the final anomaly map. All of these stages are inherited from the $G^{2}$SF-style pipeline; NTS is exclusively the added top-*q* term in the training objective of Equation (12), and it leaves the deployed inference path unchanged.

The main innovation is NTS, which directly regularizes high-response trainer-labeled normal fused-score entries used as training-time proxies. The primary implementation uses a batchwise tail estimator. For each mini-batch, NTS estimates a top-*q* threshold from the current normal fused scores, selects entries in the resulting upper tail, and penalizes their mean response. The threshold selection does not receive gradients, while gradients propagate through the selected current-batch scores to update the fusion scorer. This batchwise design requires no cross-batch estimator state. An overview of the proposed framework is illustrated in Figure 1.

### 3.2. Problem Setting

Let an input sample be denoted by

(1) $$x=\left(x^{\mathrm{rgb}},x^{3d}\right),$$

where $x^{\mathrm{rgb}}$ and $x^{3d}$ are the image and point cloud, respectively. The detector outputs a spatial anomaly score map

(2) $$S\left(x\right)\in R^{H\times W},$$

where $S_{u,v}\left(x\right)$ indicates the anomaly evidence at spatial location (u,v).

### 3.3. Base Fusion Objective

The reproduced trainer used in this study retains the complete nonzero objective executed by the reproduced $G^{2}$SF-style fusion trainer. It follows a similar discrimination-and-geometry decomposition, but its individual terms are not equation-by-equation identical to the original $G^{2}$SF formulation [5]; no equation-level identity is claimed. In the main text, we introduce only the executed fused training score, the trainer label sets, and the aggregate pre-NTS base objective.

Let V={rgb,3d} denote the modalities, $d_{i,m}^{\left(j\right)}$ the normalized modality retrieval distance, $w_{i,m}^{\left(j\right)}$ the local scale, and $g_{m}$ the global modality weight. The neighbor-level fused score and mean modality distance are

(3) $$s_{i}^{\left(j\right)}=\sum_{m\in V}w_{i,m}^{\left(j\right)}g_{m}d_{i,m}^{\left(j\right)},\;{\bar{d}}_{i}^{\left(j\right)}=\frac{1}{\left|V\right|}\sum_{m\in V}d_{i,m}^{\left(j\right)}.$$

The executed training route uses the first independently retrieved neighbor pair,

(4) $$z_{i}=s_{i}^{\left(0\right)},$$

whereas deployed inference aggregates multiple neighbors as $s_{i}=\min_{j}s_{i}^{\left(j\right)}$. Thus, $z_{i}$ is a training proxy rather than the final deployed patch score.

For a mini-batch B, the trainer label is $\ell_{i}\in \left\{+1,-1\right\}$, where +1 denotes a normal foreground patch and −1 denotes a pseudo-anomaly patch. Define

(5) $$N=\left\{i\in B:\ell_{i}=+1\right\},\;A=\left\{i\in B:\ell_{i}=-1\right\},\;B=\left|B\right|=\left|N\right|+|A|.$$

With these definitions, the executed pre-NTS base objective is

(6) $$\begin{array}{cc} L_{\mathrm{base}}\left(N,A\right)= & \lambda_{\mathrm{svdd}}L_{\mathrm{SVDD}}^{\mathrm{wt}}+\lambda_{\mathrm{margin}}L_{\mathrm{margin}}+\lambda_{\mathrm{scale}}L_{\mathrm{scale}}\\ \; & +\lambda_{\mathrm{perm}}L_{\mathrm{perm}}+\lambda_{\mathrm{cons}}L_{\mathrm{cons}}+\lambda_{1}L_{1}. \end{array}$$

The base objective uses both the normal and pseudo-anomaly sets defined in Equation (5). The full definitions of its component losses, their implementation conditions, and the inactive compatibility components are provided in Appendix A. The base objective uses both normal and pseudo-anomaly entries, whereas NTS adds extra gradients only through selected high-response normal entries.

### 3.4. Normal Tail Suppression

False positives at a low evaluation FPR arise from high-ranked normal evaluation scores. NTS uses the top-*q* current-batch normal training scores as a proxy for such responses and penalizes their mean. Here, *q* is only the selected fraction of trainer-labeled normal scores in a mini-batch; it does not directly specify the test-time pixel FPR. Because the threshold is detached, selection carries no gradient and the penalty acts only through the selected entries. Transfer from this training proxy to final pixel maps is empirical rather than guaranteed and is examined descriptively in Section 4.9.

For mini-batch $B_{t}$, define $N_{t}=\left\{i\in B_{t}:\ell_{i}=+1\right\}$, $A_{t}=\left\{i\in B_{t}:\ell_{i}=-1\right\}$, and $N_{t}=\left|N_{t}\right|$. Assume that a valid batch contains at least one normal entry, so $N_{t}\geq 1$. Using the executed training score $z_{i}$ from Equation (4), let the current-iteration normal fused patch-level scores be denoted by

(7) $$S_{t}={\left\{s_{t,i}^{N}\right\}}_{i=1}^{N_{t}}=\left\{z_{i}:i\in N_{t}\right\}.$$

The fusion dataset labels dilated pseudo-anomaly mask patches as anomalous and all remaining foreground patches as normal. A normal-score entry is the fused score of one such foreground patch with trainer label +1; pseudo-anomaly entries use label −1. For the primary batchwise NTS implementation, *q* is clamped to $\left[1/N_{t},1\right]$ and the selected-rank count is

(8) $$k_{t}=\left\lceil qN_{t}\right\rceil .$$

Let ${\mathrm{TopK}}_{k_{t}}$ return the $k_{t}$ largest entries. The detached threshold is

(9) $$\theta_{t}^{\mathrm{batch}}=\min{\mathrm{TopK}}_{k_{t}}\left(\mathrm{sg}(S_{t})\right),$$

where sg(·) denotes stop-gradient/detach. The selected current-batch high-response normal entries are

(10) $$I_{t}=\left\{i|s_{t,i}^{N}\geq \theta_{t}^{\mathrm{batch}}\right\},$$

and the NTS loss is

(11) $$L_{\mathrm{NTS}}^{\left(t\right)}=\frac{1}{|I_{t}|}\sum_{i\in I_{t}}s_{t,i}^{N}.$$

Because selection uses “≥”, ties at the threshold may yield more than $k_{t}$ entries. For the primary batchwise NTS implementation, the selected set cannot be empty when $N_{t}\geq 1$. If a batch has no normal entry, the implementation returns zero NTS loss and does not update estimator state. This formulation makes the optimization target explicit: NTS regularizes current-batch normal training entries in the upper tail of the fused-score distribution.

The NTS-augmented training objective combines the base loss with the normal-tail penalty:

(12) $$L^{\left(t\right)}\left(N_{t},A_{t}\right)=L_{\mathrm{base}}\left(N_{t},A_{t}\right)+\lambda_{\mathrm{tail}}L_{\mathrm{NTS}}^{\left(t\right)}\left(N_{t}\right).$$

The base objective uses both normal and pseudo-anomaly entries, whereas NTS adds extra gradients only through the selected high-response entries in $N_{t}$.

### 3.5. Training, Fixed-Final Checkpointing, and Inference

During training, frozen RGB and point-cloud features and their nearest coreset neighbors form a pseudo-anomaly fusion dataset. The generator uses transformed nominal samples as donors, foreground-restricted Perlin masks, and cut-and-paste synthesis; its donor list contains nominal point clouds from the ten training categories. For the primary configuration, independent nonnegative perturbations are applied to the patch and nearest-prototype features before Equations (3)–(6) are evaluated; each modality’s perturbation magnitude is sampled up to 0.1 times its stored mean score. NTS acts only during fusion-stage optimization after the fused training scores are computed.

Neither arm is trained from scratch. For each category, both arms start from the same per-category checkpoint released in the official $G^{2}$SF GitHub repository [22], which was archived locally before the experiments. The pseudo-fusion cache is generated once from this common initialization and then shared byte-identically between the no-NTS and NTS arms. Both arms are continued for E=15 additional epochs and evaluated using the checkpoint saved after the final epoch (zero-based epoch 14). No training or validation loss is used to rank epochs. This fixed-final rule is identical for the no-NTS and NTS arms, and the only objective-affecting difference is setting $\lambda_{\mathrm{tail}}=0$ or 1 in Equation (12). During inference, the model uses unperturbed frozen backbones and memory-bank retrieval, with no NTS selection, state, or loss. The final patch score $\min_{0\leq j\leq K}s_{i}^{\left(j\right)}$ is bilinearly upsampled to 224×224 and smoothed with the released radius-4 Gaussian filter. The retained benchmark artifacts are then min–max normalized per score branch over the evaluation set. This positive affine transformation preserves ranking-based area under the receiver operating characteristic curve (AUROC), per-region overlap (PRO), and AUPRO values; an online deployment can instead retain raw scores or use a fixed calibration-set transform.

### 3.6. Algorithmic Summary

Algorithm 1 summarizes the complete NTS procedure with the batchwise tail estimator. The tail set is recomputed independently for every training mini-batch, and the final epoch is saved without adaptive checkpoint selection.
**Algorithm 1:** Fusion-stage training and inference with NTS**Require:** 
Frozen RGB/3D features and memory banks; pseudo-anomaly fusion data; trainer labels $\ell_{i}\in \left\{+1,-1\right\}$ (+1: normal); tail fraction *q*; weight $\lambda_{\mathrm{tail}}$; epoch count *E*1:Initialize the fusion scorer and global modality weights**Training**2:**for** e=1,…,E **do**3:    **for** mini-batch B **do**4:        Compute perturbed training scores $z_{i}=s_{i}^{\left(0\right)}$ and the base loss $L_{\mathrm{base}}$5:        $N\leftarrow \{i\in B:\ell_{i}=+1\}$6:        **if** |N|=0 **then**7:           $L_{\mathrm{NTS}}\leftarrow 0$8:        **else**9:             $\bar{q}\leftarrow \min\{1,\max\{q,1/\left|N|\}\right\}$; $k_{q}\leftarrow \lceil \bar{q}\left|N|\right\rceil$10:           $\theta \leftarrow \min{\mathrm{TopK}}_{k_{q}}\;\left(\mathrm{sg}(\left\{z_{i}:i\in N\right\})\right)$11:           $I\leftarrow \{i\in N:z_{i}\geq \theta \}$12:           $L_{\mathrm{NTS}}\leftarrow {\left|I\right|}^{-1}\sum_{i\in I}z_{i}$13:        **end if**14:        $L\leftarrow L_{\mathrm{base}}+\lambda_{\mathrm{tail}}L_{\mathrm{NTS}}$15:        Update the fusion scorer and global modality weights using ∇L16:    **end for**17:    **if** e=E **then**18:        Save the final checkpoint and global scale19:    **end if**20:**end for****Inference**21:Load the fixed-final checkpoint and global scale22:**for** test sample *x* **do**23:    Compute neighbor scores $s_{i}^{\left(j\right)}$ and patch scores $s_{i}\leftarrow \min_{0\leq j\leq K}s_{i}^{\left(j\right)}$24:    Bilinearly upsample to 224×224 and apply the released radius-4 Gaussian smoothing25:**end for**26:Apply the retained branch-wise normalization for benchmark reporting

## 4. Experiments

### 4.1. Experimental Setup

#### 4.1.1. Dataset

Experiments primarily use MVTec 3D-AD [1], a widely used standard benchmark for RGB–3D industrial anomaly detection. It provides aligned RGB observations and industrial-sensor 3D scans from ten real industrial object categories, enabling rigorous RGB–3D correspondence and direct comparison with established methods. Training excludes test data and real anomalies. Following the released $G^{2}$SF implementation [22], pseudo anomalies may use nominal point-cloud donors from other training categories; thus, the fusion data are normal-only but not strictly class-specific.

#### 4.1.2. Evaluation Metrics

We report I-AUROC, P-AUROC, and AUPRO@30% as conventional metrics [1,5,8,15]. The primary metric is mean AUPRO@1% over ten categories. Primary values use the released $G^{2}$SF evaluator with 100 thresholds to preserve implementation-level comparability [22]. AUPRO@α conceptually denotes the normalized area under the PRO–FPR curve up to FPR α.

For category *c*, let $\Omega_{c}$ be all locations in its complete 224×224 test maps, let $g_{c,p}\in \left\{0,1\right\}$ be the test ground-truth (GT) mask with $g_{c,p}=1$ denoting an anomalous pixel, and let $S_{c,p}$ be the corresponding normalized fusion score. Define $\Omega_{N,c}=\left\{p\in \Omega_{c}:g_{c,p}=0\right\}$. The category-level false-positive rate at threshold τ is

(13) $${\mathrm{FPR}}_{c}\left(\tau \right)=\frac{\sum_{p\in \Omega_{N,c}}I\left[S_{c,p}>\tau \right]}{|\Omega_{N,c}|}.$$

Following the region-level evaluation introduced with MVTec Anomaly Detection (MVTec AD) [8], for category *c*, ${\mathrm{PRO}}_{c}\left(f\right)$ is the mean overlap over its 8-connected GT anomalous regions at false-positive rate *f*. For a low-FPR integration limit α, the classwise AUPRO is

(14) $${\mathrm{AUPRO}}_{c}@\alpha =\frac{1}{\alpha }\int_{0}^{\alpha }{\mathrm{PRO}}_{c}\left(f\right)\;df.$$

The final reported score is the mean over all *C* categories:

(15) $$\mathrm{AUPRO}@\alpha =\frac{1}{C}\sum_{c=1}^{C}{\mathrm{AUPRO}}_{c}@\alpha .$$

In the main low-FPR evaluation, α=0.01, corresponding to AUPRO@1%.

Following the released $G^{2}$SF implementation [22], the evaluator is invoked independently for each category *c* on its complete normalized maps. It sorts the $N_{c}=\left|\Omega_{N,c}\right|$ GT-normal pixel scores, samples order-statistic positions over the full normal-score range, assigns sampled rank *r* the value $f_{c,r}=1-\left(r+1\right)/N_{c}$, and applies trapezoidal integration with linear interpolation at f=0.01. At 100 sampled positions, the 0–1% interval is sparsely resolved. When scores are tied, the implementation retains the rank-position convention rather than recounting strict threshold exceedances in Equation (13); region overlap at a sampled threshold uses the strict score-above-threshold rule. All reported AUPRO@1% means are taken directly from the retained evaluator pipeline output files; for the fixed-final matched pair, these pipeline outputs are 47.142% without NTS and 47.494% with NTS-Batch.

#### 4.1.3. Implementation

The primary comparison consists of matched local continuation without NTS and matched local continuation with NTS using the batchwise tail estimator, both built on the same reproduced $G^{2}$SF-style continuation route. They share frozen DINO ViT-B/8 [23] and PointMAE [24] backbones, byte-identical pseudo-fusion cache, initialization, data ordering, training schedule, optimizers, fixed-final checkpoint rule, deployed inference architecture, and evaluator. Fusion training uses Adam with learning rate $1.5\times 10^{-4}$ and weight decay $10^{-4}$; the two global modality weights use Adam with learning rate $5\times 10^{-3}$ and the same weight decay. The fixed base-loss coefficients shared by both arms were $\left(\lambda_{\mathrm{svdd}},\lambda_{\mathrm{margin}},\lambda_{\mathrm{scale}},\lambda_{\mathrm{perm}},\lambda_{\mathrm{cons}},\lambda_{1}\right)=\left(1,10,3,20,40,5\times 10^{-4}\right)$. Training uses batch cap 8192. The primary NTS configuration uses the current-batch quantile with q=0.02 and $\lambda_{\mathrm{tail}}=1.0$; the control sets $\lambda_{\mathrm{tail}}=0$, which is the only objective-affecting change. The arm-specific tail-estimator trace/save flags only serialize diagnostics and do not enter optimization or inference. The primary matched pair uses seed 0. To estimate run-to-run variability, we additionally repeated both arms with seeds 1–4; Section 4.5 reports all five paired realizations and the uncertainty interval for their paired differences. The main pair used Python 3.11.14, PyTorch 2.11.0, Lightning 2.6.0, and timm 0.9.12 with CUDA 12.8 on one NVIDIA GeForce RTX 5090 D v2 GPU (NVIDIA Corporation, Santa Clara, CA, USA). NTS adds no trainable parameters, modules, or state to the deployed inference path. During training, its additional computation consists of a top-*q* selection over the current-batch normal scores followed by a mean over the selected entries.

The primary comparison is a matched continuation experiment rather than a from-scratch $G^{2}$SF reproduction. Both arms start from the same per-category checkpoints released in the official $G^{2}$SF repository [22] and share the same locally generated pseudo-fusion cache before the identical 15-epoch continuation. The checkpoint saved after the final epoch (zero-based epoch 14) is evaluated under the fixed-final rule without adaptive selection.

### 4.2. Published Context and Matched Local Comparison

Table 1 separates published contextual results from the matched local comparison. $G^{2}$SF reports 46.8% AUPRO@1% in the published comparison [5], while the official repository separately reports 47.1% for its released retrained weights [22]. The present NTS result of 47.494% is numerically higher than both values. However, these results were obtained under different training and reporting identities and are therefore treated only as non-matched context rather than evidence of state-of-the-art superiority. Within the matched local pair, the directly attributable NTS difference is +0.352 percentage points over the no-NTS control (47.142%).

The NTS loss configuration does not produce uniformly higher values across all metrics. Within Panel B, it has an observed +0.352 percentage-point (pp) higher evaluator-compatible low-FPR value, while P-AUROC and AUPRO@30% change by only +0.017 pp and +0.036 pp, respectively, and I-AUROC decreases by 0.482 pp. Because checkpointing and all other paired settings are identical, this fixed-seed difference isolates the configured NTS loss intervention within the reproduced continuation protocol. The observed low-FPR difference remains relevant to industrial inspection, where reliable defect localization under stringent false-positive constraints is often more important than uniform improvements across broad-range metrics.

### 4.3. Classwise Analysis

Table 2 reports the complete ten-category paired comparison. The paired differences in dataset order are −0.20, +0.24, +0.42, +0.81, −0.10, +0.71, +1.17, +0.10, −0.19, and +0.56 pp. Peach shows the largest increase, followed by Cookie and Foam; Bagel, Dowel, and Rope decrease slightly. Thus, seven categories increase and three decrease, indicating category-dependent response to a single tail proportion and weight. Texture, missing-point patterns, and fusion-score distributions are plausible contributors, but this study does not separately identify those causes or support category-level significance inference. Figure 2 illustrates retained anomaly cases from three categories with positive changes and three with negative changes.

### 4.4. Post Hoc Normal-Tail Operating-Point Analysis

As a post hoc descriptive analysis, we examined whether the fixed-final fused maps exhibit behavior consistent with the targeted low-FPR operating regime. NTS is optimized on patch-level training proxies, whereas this analysis uses final test-time pixel maps. Moreover, test ground-truth labels are used to identify normal pixels and to determine the empirical normal-score threshold for each configuration. This analysis is therefore neither a deployable calibration procedure nor a causal mechanism test. When PRO was evaluated on the complete normalized maps at the threshold defined by the 99th percentile of each configuration’s test GT-normal pixel scores, NTS yielded a higher value in nine of ten categories, with a macro-average NTS-minus-control difference of +0.324 percentage points. This single-operating-point observation is consistent with, but does not establish, the intended tail-focused behavior and remains distinct from the integrated AUPRO@1% metric. Figure 3 shows representative defect-free test samples and their corresponding score maps.

### 4.5. Multi-Seed Reproducibility

To assess run-to-run variability, we repeated the complete no-NTS and NTS-Batch arms for four additional random seeds (seeds 1–4) under the identical fixed-final protocol, yielding five paired realizations in total. Table 3 reports the per-seed pipeline-reported AUPRO@1% and paired difference. Treating the five seed-level paired differences as the analysis units gives a mean of +0.228 percentage points, sample standard deviation of 0.158 pp, and paired *t* 95% confidence interval (CI) of [+0.032,+0.423] pp. All five differences are positive, supporting a positive mean trend within this MVTec continuation protocol.

### 4.6. Threshold-Resolution Robustness

The released $G^{2}$SF evaluator samples 100 order statistics, which resolves the 0–1% FPR interval sparsely. We replayed the same seed-0 control and NTS prediction maps with the same ground truth and evaluator at 100, 500, 1000, and 5000 thresholds, changing only the number of thresholds. Table 4 shows a positive paired difference at all four tested resolutions, although both absolute values and paired differences depend on threshold resolution. The primary pipeline value of 47.494% is the mean of classwise values retained at four-decimal precision (+0.352 pp), whereas the full-map 100-threshold replay gives 47.495% (+0.353 pp) from full-precision values (47.141720% and 47.494779%).

We retain 100 thresholds for the primary comparison because this is the released $G^{2}$SF evaluator setting and preserves implementation-level comparability with the retained pipeline. The higher-threshold replay is used only as a robustness check. As Table 4 shows, the NTS-minus-control sign remains positive at all tested resolutions, while absolute values and effect magnitude are threshold-dependent. All 100-threshold AUPRO@1% values are therefore interpreted as protocol-specific paired comparisons.

### 4.7. Sensitivity Analysis

The primary settings q=0.02, $\lambda_{\mathrm{tail}}=1.0$, and 15 epochs were fixed before the final matched runs based on prior experience; they were not selected by independent validation. Table 5 is therefore a sensitivity analysis, not validation-based model selection.

We evaluated the tail fraction *q* and tail weight $\lambda_{\mathrm{tail}}$ separately under the same fixed-final protocol and byte-identical seed-0 pseudo-fusion cache. Table 5 reports the measured values. The best observed tail fraction is q=0.02, whereas performance declines at larger fractions. For the tested weights, $\lambda_{\mathrm{tail}}=2.0$ gives the highest value, closely followed by the primary setting of 1.0. These results characterize sensitivity within the measured configuration rather than independent validation-based parameter selection; the training fraction *q* does not directly set the test-time pixel FPR.

### 4.8. Control Ablations

We compared batchwise top-*q* NTS with mean-all, a size-matched random subset, maximum-only, a CVaR-style hinge, and cross-batch first-in, first-out (FIFO) under the same seed-0 cache and fixed-final protocol (Table 6). Mean-all and random-subset penalties underperform the no-NTS control, whereas all four tail-focused variants exceed it. These results support targeting the normal-score tail, with batchwise top-*q* achieving the highest observed AUPRO@1% among the tested variants.

### 4.9. Pixel-Level Score-Distribution Analysis

For the fixed seed-0 matched pair, we restored the raw fused scores using each arm’s stored min–max range. Baseline GT-normal p99, p99.5, and p99.9 thresholds (the 99th, 99.5th, and 99.9th percentiles, respectively) were then applied unchanged to both arms. Table 7 reports the requested macro-averaged normal-tail statistics and false-positive (FP) counts. The Change column reports percentage changes relative to the no-NTS baseline. FP pixels per image changed by +9.3%, −3.1%, and −20.8% at p99, p99.5, and p99.9, respectively. The effect is therefore threshold-dependent and does not establish a uniform reduction in false positives. Because test ground truth defines the normal pixels and thresholds, this analysis is post hoc and descriptive rather than a deployment calibration or an independent mechanism test.

### 4.10. Transfer to an Additional Map-Fusion Configuration

We additionally evaluated NTS on frozen anomaly maps from a separately developed, unpublished DAMD-derived RGB–3D pipeline developed by the same authors; the companion manuscript is under review. The evaluated positive-linear head (two softplus-constrained modality weights and one bias per category) is an experimental probe, not an official DAMD component or established external baseline. Before test-map extraction, we fixed q=0.02, $\lambda_{\mathrm{tail}}=1.0$, five paired seeds, all ten MVTec 3D-AD categories, and ten-category macro AUPRO@1% with 100 thresholds. Control and NTS otherwise share the same frozen maps, initialization, binary-cross-entropy objective, data order, 200-epoch schedule, batch size 4096, 512 normal patch locations per training map, sampled without replacement using deterministic random sampling, and evaluation code.

The results are summarized in Table 8. NTS increases AUPRO@1% from 37.528% to 44.087% (+6.559 pp; paired 95% CI [+6.548,+6.571] pp), with positive differences for all five seeds. Eight of ten categories improve, including a small increase for Tire; Dowel and Rope decrease. These results extend the positive evidence for NTS to a second RGB–3D fusion configuration on the complete ten-category MVTec 3D-AD benchmark. The additional experiment evaluates a frozen-map fusion head rather than end-to-end DAMD; broader generalization limits are discussed in Section 5.2.

## 5. Discussion

The fixed-final MVTec paired results show that adding the NTS loss changes performance most clearly in the stringent low-FPR localization region. This observed difference is relevant to industrial inspection under strict false-positive constraints, although its magnitude and direction vary across metrics and object categories. The positive DAMD-derived probe extends the evidence to one additional experimental map-fusion configuration, but its unpublished same-author origin and restricted probe design preclude an independent architecture-general claim.

### 5.1. Performance Trade-Offs and Category Dependence

The NTS loss does not produce uniformly higher values across all metrics, and its higher low-FPR value is accompanied by only marginal changes in P-AUROC and AUPRO@30%. The classwise response is not uniform. Bagel, Dowel, and Rope decrease in AUPRO@1%, although their top-1% raw normal-tail means fall by approximately 7.20%, 1.84%, and 6.07%, respectively. These declines therefore cannot be explained simply by a failure to suppress the normal tail. Category-specific geometry, missing-point patterns, and fused-score distributions are plausible contributors, but are not isolated by the present experiments. The concurrent 0.482-pp decrease in I-AUROC is likewise treated as an unresolved trade-off.

### 5.2. Limitations

Fixed-setting evaluations on Real-IAD D3 [25] and EyeCandies [26] did not improve AUPRO@1% over their matched controls. They therefore do not establish dataset-independent robustness and indicate that fixed *q* and $\lambda_{\mathrm{tail}}$ are distribution-sensitive. The post hoc operating-point analysis of Section 4.4 uses test ground-truth labels to identify normal pixels and to set its threshold; it is a descriptive analysis only and is not a deployable calibration procedure, nor an independent causal validation of the mechanism. The primary 100-threshold AUPRO@1% values are retained for implementation-level comparability with the released $G^{2}$SF evaluator; absolute values are threshold-resolution dependent and should not be compared across threshold counts as replacement primary protocols. The fixed-final protocol removes adaptive checkpoint-selection bias but may miss a better epoch for either arm. Moreover, NTS optimizes patch-level training scores as proxies for final pixel-level predictions and therefore provides no formal guarantee on the test-time FPR. The evidence covers one established G^2^SF-style pipeline and one unpublished experimental DAMD-derived map-fusion probe on MVTec 3D-AD; broader cross-backbone, cross-fusion, and cross-dataset generality remains unverified. The 15-epoch continuation from the official released per-category $G^{2}$SF checkpoints means the main result characterizes NTS within this specific reproduction and continuation-training protocol rather than a from-scratch claim about $G^{2}$SF. Future work should estimate the tail setting from training-only normal statistics or a held-out validation split and evaluate multiple established backbones and fusion methods. The current evaluation focuses on pixel-level localization rather than sample-level production outcomes, which remain to be investigated in practical settings. The DAMD-derived evidence is limited to the experimental frozen-map probe: a prior score-fusion probe at a different endpoint was negative, and prior sample-level outputs were known, so no human-blindness claim is made. Sealed map parts permit byte-identical metric replay, but the raw test RGB/point-cloud/ground-truth bytes were not pre-hashed. Section 4.9 is likewise post hoc because test ground truth defines its normal pixels and raw thresholds; it is not deployment calibration or an independent mechanism test.

## 6. Conclusions

This paper studied Normal Tail Suppression (NTS), a top-*q* regularizer for high-response normal fused-score entries during RGB–3D fusion training. Under an identical 15-epoch fixed-final protocol, the retained MVTec evaluator pipeline reports a 47.142% mean AUPRO@1% without NTS and 47.494% with the primary batchwise NTS loss, which is an observed fixed-seed difference of +0.352 pp; seven of ten categories increase. Across five MVTec seed-level paired differences, the mean is +0.23 pp (95% CI [+0.03,+0.42] pp), a positive trend within this continuation protocol. Seed-0 controls show that maximum-only, CVaR, FIFO, and batchwise top-*q* variants all exceed the control, with batchwise top-*q* giving the highest observed value among those measured. A separately frozen DAMD-derived map-fusion probe yields a +6.559 pp ten-category macro AUPRO@1% difference on MVTec 3D-AD, providing bounded configuration-transfer evidence. Fixed-setting cross-dataset evidence does not support a dataset-independent claim, and official end-to-end, cross-backbone, and broader cross-fusion transfer remain unestablished.

## Figures and Tables

**Figure 1:**
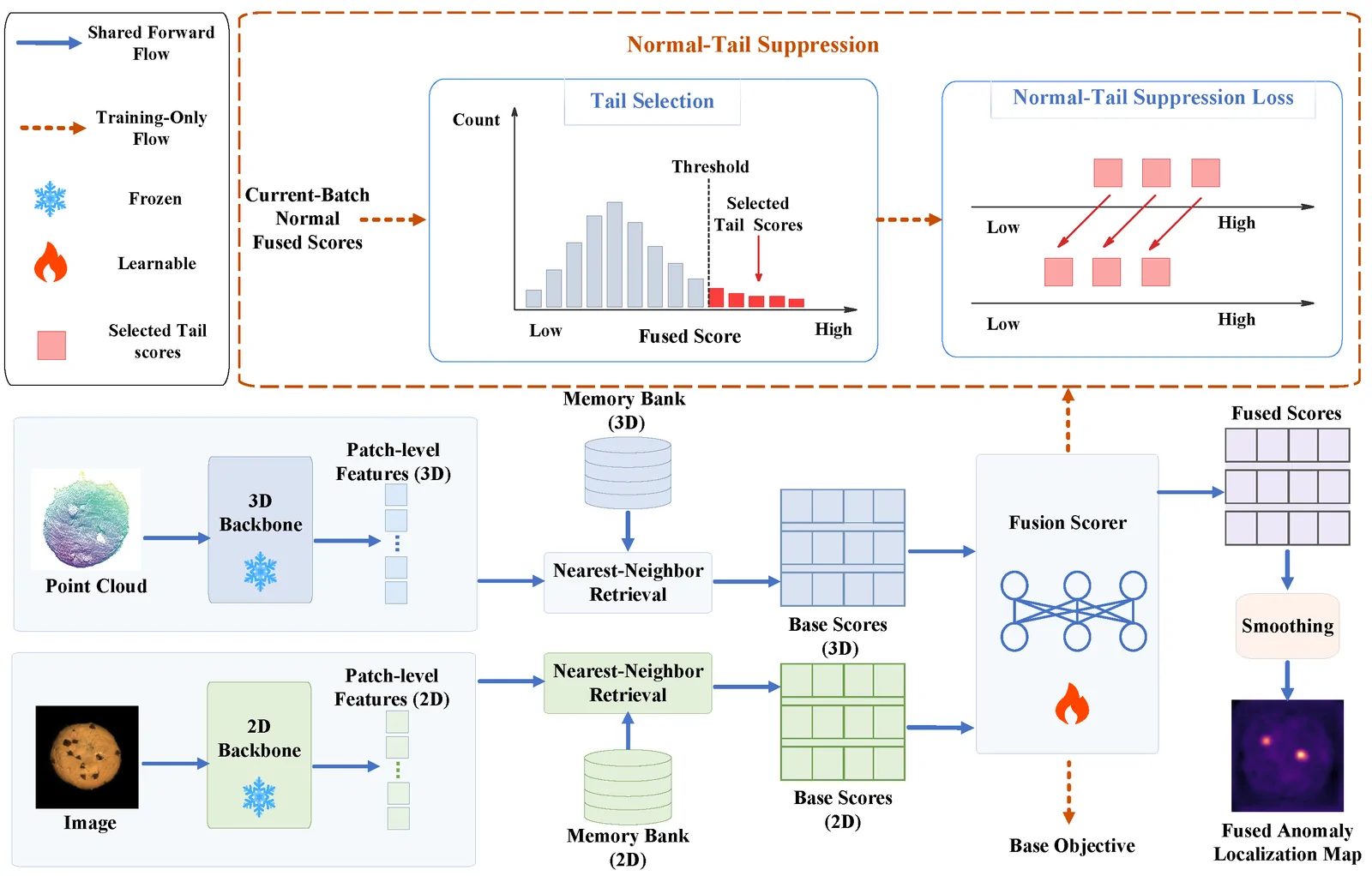
Overview of NTS. A $G^{2}$SF-style RGB–3D pipeline produces fused patch scores from appearance and geometric anomaly evidence. During training, the primary NTS implementation uses a batchwise tail estimator to apply a top-*q* penalty to high-response trainer-labeled normal fused-score entries, whereas deployment omits NTS and retains the original inference path.

**Figure 2:**
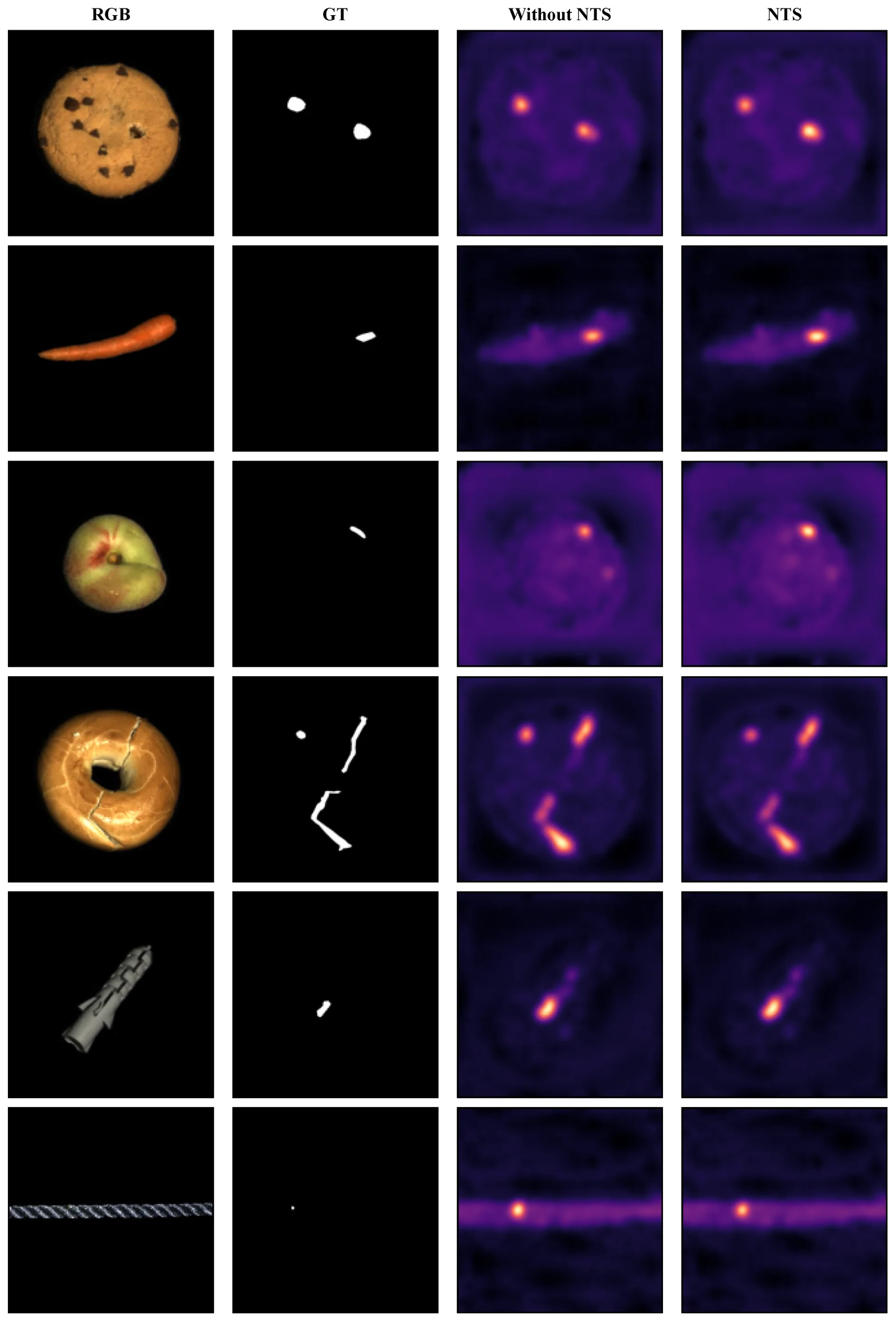
Qualitative examples from the matched fixed-final predictions. The first three rows correspond to categories with positive AUPRO@1% changes (Cookie, Carrot, and Peach), whereas the last three correspond to categories with negative changes (Bagel, Dowel, and Rope). In the score maps, dark purple indicates lower anomaly scores and bright yellow indicates higher anomaly scores; the control and NTS maps within each row use the same display range. In the ground-truth masks, white indicates anomalous pixels and black indicates normal pixels.

**Figure 3:**
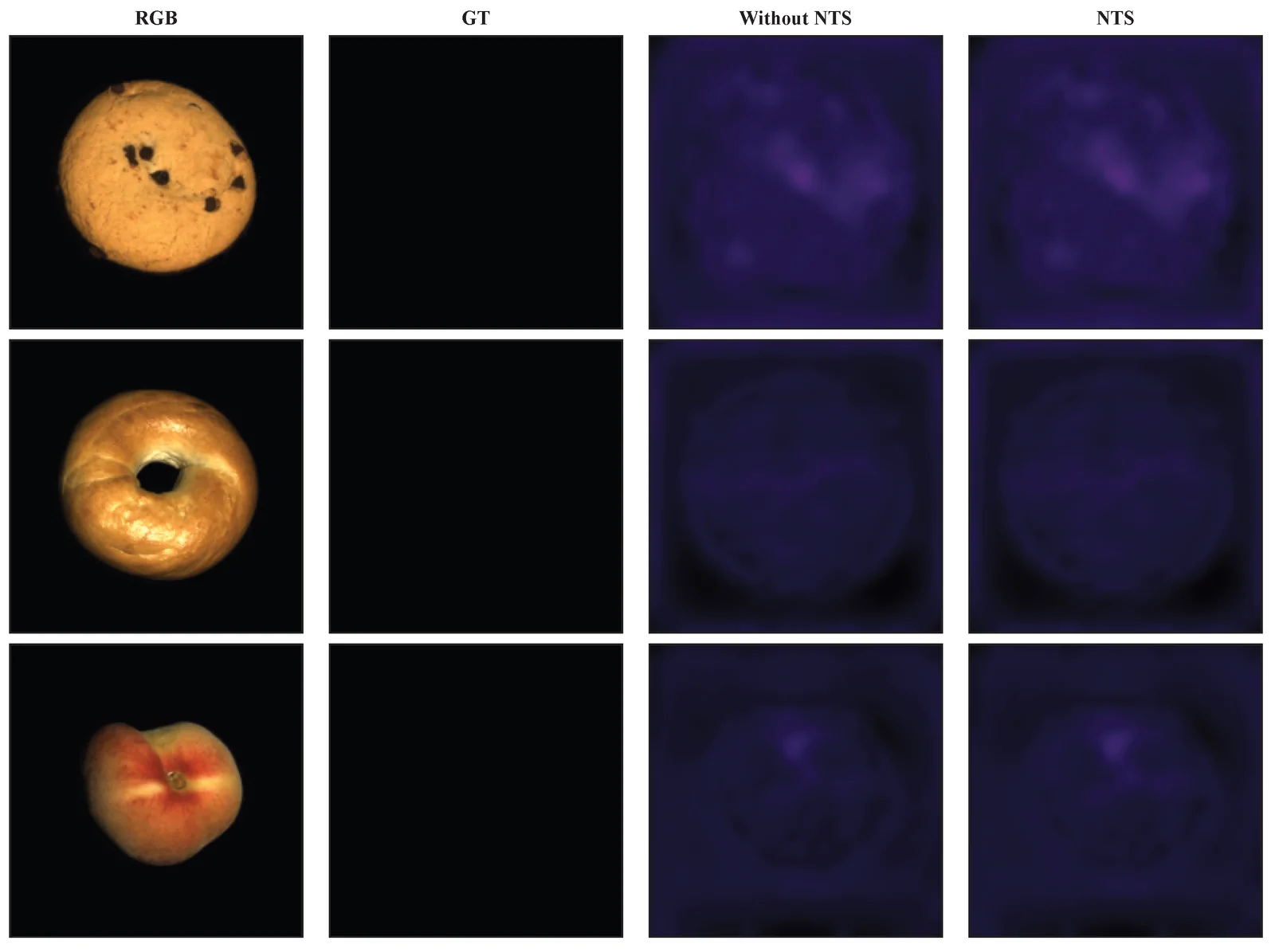
Defect-free (“good”) test samples. Each row shows the RGB image, ground-truth mask, and matched control and NTS score maps. In the score maps, dark purple indicates lower anomaly scores and bright yellow indicates higher anomaly scores. For each category, the control and NTS maps share the same raw-score display range computed over all retained test maps from both arms. In the ground-truth masks, black indicates normal pixels and white would indicate anomalous pixels; all displayed samples are defect-free. These examples visualize changes in normal-region responses but do not by themselves establish an overall false-positive improvement.

**Table 1:** Published context and matched local comparison on MVTec 3D-AD. Panel A values are contextual percentages transcribed from the published $G^{2}$SF comparison [5] at its reported precision and are not matched to Panel B. Citations in the Panel A method labels identify the corresponding original methods, whereas [5] is the source of the reported comparison values. Panel B reports the complete local pair; bold indicates the higher value only within each panel. The last column reports the retained pipeline outputs from the released $G^{2}$SF evaluator with 100 thresholds [22]. For the matched local pair in Panel B, AUPRO@1% is computed from the fused RGB–3D score branch.

Method	I-AUROC	P-AUROC	AUPRO@30%	AUPRO@1%
Panel A: Published contextual results				
AST [6]	93.7	97.6	94.4	39.8
CFM [4]	95.4	99.3	97.1	45.5
Shape-guided [17]	94.7	99.6	97.6	45.6
$G^{2}$SF [5]	**97.1**	**99.7**	**97.9**	**46.8**
Panel B: Matched local continuation				
Without NTS	**97.527**	99.738	97.934	47.142
With NTS (batchwise, ours)	97.045	**99.755**	**97.970**	**47.494**

**Table 2:** Classwise AUPRO@1% (%) for the matched local pair using the released $G^{2}$SF evaluator with 100 thresholds. Classwise entries are displayed to two decimal places, and the final column gives the retained pipeline mean to three decimal places. Bold indicates the higher value within each category.

Configuration	Bagel	Cable_Gland	Carrot	Cookie	Dowel	Foam	Peach	Potato	Rope	Tire	Mean
Matched continuation without NTS	**48.16**	44.12	48.86	48.58	**41.23**	46.94	49.23	49.83	**47.97**	46.50	47.142
Matched continuation with NTS (batchwise)	47.96	**44.36**	**49.28**	**49.39**	41.13	**47.65**	**50.40**	**49.93**	47.78	**47.06**	**47.494**

**Table 3:** Paired mean AUPRO@1% (%) across five fixed-final seeds (100 thresholds). Each seed is one matched training realization of both arms; the paired difference is NTS minus no-NTS.

	Seed 0	Seed 1	Seed 2	Seed 3	Seed 4	Mean ± Std
Without NTS	47.142	47.342	46.871	47.449	47.386	47.238 ± 0.235
With NTS	47.494	47.475	47.311	47.569	47.479	47.466 ± 0.094
Difference	+0.352	+0.133	+0.440	+0.120	+0.093	+0.228 ± 0.158

**Table 4:** Mean AUPRO@1% (%) replayed at increasing threshold counts on the fixed-final seed-0 maps. The 100-threshold row follows the same classwise pipeline reporting convention as Table 1; the 500/1000/5000 rows are full-map replays.

Thresholds	Without NTS	With NTS	Difference
100	47.142	47.494	+0.352
500	76.295	76.755	+0.460
1000	78.365	78.879	+0.514
5000	79.301	79.853	+0.552

**Table 5:** Sensitivity of mean AUPRO@1% (%) to the tail fraction *q* and tail weight $\lambda_{\mathrm{tail}}$ (seed 0, 100 thresholds).

Panel A: Tail fraction with $\lambda_{\mathrm{tail}}=1.0$				
*q*	0.01	0.02	0.05	0.10
AUPRO@1%	47.144	47.494	46.942	45.321
Panel B: Tail weight with q=0.02				
$\lambda_{\mathrm{tail}}$		0.5	1.0	2.0
AUPRO@1%		47.254	47.494	47.522

**Table 6:** Control ablations: mean AUPRO@1% (%) for alternative tail penalties (seed 0, 100 thresholds). The no-NTS control is 47.142%; bold indicates the highest value among the tested configurations.

Configuration	AUPRO@1%
Without NTS	47.142
NTS (top-*q*, q=0.02)	**47.494**
Mean of all normal (q=1.0)	45.969
Random subset (q=0.02)	46.022
Maximum score only	47.232
CVaR-style hinge (0.98 quantile)	47.214
Cross-batch FIFO	47.347

**Table 7:** Macro-averaged raw (de-normalized) normal-score statistics over ten categories.

Statistic	Without NTS	With NTS	Change
Normal top-1% mean	1.301	1.266	−2.7%
Normal p99	0.896	0.903	+0.8%
Normal p99.5	1.152	1.131	−1.8%
Normal p99.9	1.907	1.811	−5.0%
FP pixels/image @ baseline p99	500.0	546.4	+9.3%
FP pixels/image @ baseline p99.5	250.0	242.2	−3.1%
FP pixels/image @ baseline p99.9	50.0	39.6	−20.8%

**Table 8:** Protocol-frozen transfer to an experimental DAMD-derived map-fusion probe on MVTec 3D-AD. The confidence interval is paired over five training seeds on the fixed benchmark; bold indicates the higher NTS value and corresponding paired difference.

Metric	Control	NTS	Δ (pp)	95% CI (pp)	Positive Seeds
AUPRO@1%	37.528	**44.087**	** +6.559 **	[+6.548,+6.571]	5/5

## Data Availability

The datasets used in this study are MVTec 3D-AD (https://www.mvtec.com/research-teaching/datasets/mvtec-3d-ad, accessed on 19 September 2026), Real-IAD D3 (https://openaccess.thecvf.com/content/CVPR2025/html/Zhu_Real-IAD_D3_A_Real-World_2DPseudo-3D3D_Dataset_for_Industrial_Anomaly_Detection_CVPR_2025_paper.html, accessed on 19 September 2026) and EyeCandies (https://eyecan-ai.github.io/eyecandies/, accessed on 19 September 2026).

## Appendix A. Executed Base Fusion Objective

The matched clean commands route through the hybrid fusion-engine wrapper with its compatibility flag enabled. However, relation-context scaling, the learned prior, failure calibration, and tail-risk quantile regularization are all disabled in those commands. Consequently, the instantiated unified calibrator has no active subheads, its multiplicative correction is identically one, and every registered tail-risk auxiliary loss has zero contribution. The equations below therefore include every term that contributes to optimization in the reported matched pair.

Let V={rgb,3d} denote the two modalities. For patch *i*, modality m∈V, and independently retrieved neighbor index *j*, let $f_{i}^{m}$ be the frozen feature, $p_{i,m}^{\left(j\right)}$ its retrieved memory prototype, and ${\bar{d}}_{m}>0$ the corresponding mean training distance. The normalized modality distance is

(A1) $$d_{i,m}^{\left(j\right)}=\frac{{∥f_{i}^{m}-p_{i,m}^{\left(j\right)}∥}_{2}}{{\bar{d}}_{m}}.$$

Hence, $d_{i,m}^{\left(j\right)}\geq 0$. The fusion network predicts the local scale $w_{i,m}^{\left(j\right)}$, while $g_{m}\geq 0$ is the learned global modality weight with $\sum_{m\in V}g_{m}=1$.

Let ${\left[u\right]}_{+}=\max\left(u,0\right)$. Inspired by Deep SVDD [27], the reproduced scalar objective fixes the center to c=0. The corresponding squared distance is

(A2) $$D_{i}={\left(z_{i}-c\right)}^{2}=z_{i}^{2}.$$

The weighted training criterion assigns clipped score-dependent weights

(A3) $$a_{i}=\mathrm{clip}\;\left(D_{i}^{\alpha },\frac{1}{3},3\right),\;b_{i}=\mathrm{clip}\;\left({\left(D_{i}+\epsilon \right)}^{-\alpha },\frac{1}{3},3\right),$$

where the executed settings are α=2 and $\epsilon =10^{-5}$. Let

(A4) $$\rho =2\max_{i\in A_{\mathrm{train}},\;m\in V}d_{i,m}^{\left(0\right)},\;\phi_{i}={\left[\frac{1}{D_{i}+\epsilon }-\frac{1}{\rho }\right]}_{+},$$

where $A_{\mathrm{train}}$ denotes the pseudo-anomaly entries used by the adaptive-margin initialization. The executed weighted SVDD loss is the mean over the concatenated normal and pseudo-anomaly terms,

(A5) $$L_{\mathrm{SVDD}}^{\mathrm{wt}}=\frac{1}{B}\left(\sum_{i\in N}a_{i}D_{i}+\sum_{i\in A}b_{i}\phi_{i}\right).$$

The criterion separately penalizes overlap between the normal and pseudo-anomaly distance distributions. Let

(A6) $$\kappa =Q_{p}\;\left(\{D_{i}:i\in N\}\right),\;{\tilde{D}}_{i}=\frac{D_{i}}{\kappa },\;\underline{a}=\min_{i\in A}{\tilde{D}}_{i},\;n=Q_{p}\;\left(\{{\tilde{D}}_{i}:i\in N\}\right),$$

where $Q_{p}$ is the empirical *p*-quantile. The distribution-margin loss is

(A7) $$L_{\mathrm{margin}}=\frac{1}{B}\left(\sum_{i\in N}{\left[{\tilde{D}}_{i}-\underline{a}\right]}_{+}+\sum_{i\in A}{\left[n-{\tilde{D}}_{i}\right]}_{+}\right).$$

The executed setting uses p=1; therefore, when κ>0, $\kappa =\max_{i\in N}D_{i}$ and n=1.

Because the fusion head uses exp(tanh(·)), each local scale satisfies $w_{i,m}^{\left(j\right)}\in \left[e^{-1},e\right]$. The local-scale loss suppresses normal scales and pushes pseudo-anomaly scales toward the upper limit:

(A8) $$L_{\mathrm{scale}}=\frac{1}{\left|A||V\right|}\sum_{i\in A}\sum_{m\in V}\left(e-w_{i,m}^{\left(0\right)}\right)+\frac{1}{\left|N||V\right|}\sum_{i\in N}\sum_{m\in V}{\left[w_{i,m}^{\left(0\right)}-1\right]}_{+}.$$

For modality-permuted feature–prototype pairs, let ${\tilde{w}}_{i,m}^{\left(0\right)}$ denote the resulting local scales. The permutation penalty is

(A9) $$L_{\mathrm{perm}}=\frac{1}{B|V|}\sum_{i\in B}\sum_{m\in V}\left(e-{\tilde{w}}_{i,m}^{\left(0\right)}\right).$$

This term is the executed counterpart of the cross-modal mismatch constraint: breaking modality correspondence should produce large local scales.

The nearest-neighbor consistency term evaluates 2K+1 independently retrieved neighbor pairs. With K=5 and $\nu_{\mathrm{cons}}=1.2$, its proximal and distal parts are

(A10) $$\begin{array}{cc} L_{\mathrm{cons}}= & \frac{1}{KB}\sum_{j=1}^{K}\sum_{i\in B}{\left[s_{i}^{\left(j\right)}-\nu_{\mathrm{cons}}\frac{{\bar{d}}_{i}^{\left(j\right)}}{{\bar{d}}_{i}^{\left(0\right)}}s_{i}^{\left(0\right)}\right]}_{+}\\ \; & +\frac{1}{KB}\sum_{j=K+1}^{2K}\sum_{i\in B}{\left[s_{i}^{\left(0\right)}-s_{i}^{\left(j\right)}\right]}_{+}. \end{array}$$

Finally, if $W_{\mathrm{lin}}$ is the set of weight matrices in the fusion network’s linear layers, the executed regularizer is

(A11) $$L_{1}=\sum_{W\in W_{\mathrm{lin}}}{∥W∥}_{1}.$$

Biases are not included. The configured $\ell_{2}$ coefficient is zero.

Equations (A1)–(A11) apply to the executed class-balanced batches, which contain both normal and pseudo-anomaly entries. They also assume ${\bar{d}}_{m}>0$, ρ>0, κ>0, and ${\bar{d}}_{i}^{\left(0\right)}>0$ wherever the corresponding divisions are evaluated; these conditions are satisfied by the reproduced training runs.
